# Methods of Topical Administration of Drugs and Biological Active Substances for Dental Implants—A Narrative Review

**DOI:** 10.3390/antibiotics10080919

**Published:** 2021-07-28

**Authors:** Piotr Wychowański, Anna Starzyńska, Paulina Adamska, Monika Słupecka-Ziemilska, Bartosz Kamil Sobocki, Agnieszka Chmielewska, Bartłomiej Wysocki, Daniela Alterio, Giulia Marvaso, Barbara Alicja Jereczek-Fossa, Jan Kowalski

**Affiliations:** 1 Department of Oral Surgery, Medical University of Warsaw, 6 St. Binieckiego Street, 02-097 Warsaw, Poland; piotrwychowanski@wychowanski.pl; 2 Department of Oral Surgery, Medical University of Gdańsk, 7 Dębinki Street, 80-211 Gdańsk, Poland; paulina.adamska@gumed.edu.pl (P.A.); b.sobocki@gumed.edu.pl (B.K.S.); 3 Department of Human Epigenetics, Mossakowski Medical Research Center, Polish Academy of Sciences, 5 Pawińskiego Street, 02-106 Warsaw, Poland; mslupecka@imdik.pan.pl; 4 International Research Agenda 3P—Medicine Laboratory, Medical University of Gdańsk, 3a Marii Skłodowskiej-Curie Street, 80-210 Gdańsk, Poland; 5 Faculty of Material Science and Engineering, Warsaw University of Technology, 141 Wołoska Street, 02-507 Warsaw, Poland; achmielewska00@gmail.com; 6 Department of Materials Science and Engineering, The Ohio State University, 140 W 19th Ave, Columbus, OH 43210, USA; 7 Center of Digital Science and Technology, Cardinal Stefan Wyszyński University in Warsaw, Woycickiego 1/3 Street, 01-938 Warsaw, Poland; b.wysocki@uksw.edu.pl; 8 Additive Manufacturing Research Center, College of Engineering, Youngstown State University, Youngstown, OH 44555, USA; 9 Division of Radiotherapy, IEO European Institute of Oncology, IRCCS, 435 Ripamonti Street, 20141 Milan, Italy; daniela.alterio@ieo.it (D.A.); giulia.marvaso@ieo.it (G.M.); barbara.jereczek@ieo.it (B.A.J.-F.); 10 Department of Oncology and Hemato-Oncology, University of Milan, 7 Festa del Perdono Street, 20112 Milan, Italy; 11 Department of Periodontology and Oral Medicine, Medical University of Warsaw, 6 St. Binieckiego Street, 02-097 Warsaw, Poland; jkowalski@wum.edu.pl

**Keywords:** dental implants, local drug delivery, dental implant coatings, dental implant surface, medical device, dental implant construction, biocompatibility, osseointegration

## Abstract

Dental implants are, nowadays, established surgical devices for the restoration of lost teeth. Considered as an alternative for traditional prosthetic appliances, dental implants surpass them in reliability and patient feedback. Local drug delivery around the implants promotes osseointegration and reduces peri-implantitis. However, there are currently no methods of a multiple, precise topical administration of drugs to the implant area. Engineering coatings on the implants, drug application on carriers during implantation, or gingival pockets do not meet all requirements of dental surgeons. Therefore, there is a need to create porous implants and other medical devices that will allow a multiple drug delivery at a controlled dose and release profile without traumatic treatment. Due to the growing demand for the use of biologically active agents to support dental implant treatment at its various stages (implant placement, long-term use of dental superstructures, treatment of the peri-implant conditions) and due to the proven effectiveness of the topical application of pharmacological biologically active agents to the implant area, the authors would like to present a review and show the methods and devices that can be used by clinicians for local drug administration to facilitate dental implant treatment. Our review concludes that there is a need for research in the field of inventions such as new medical devices or implants with gradient solid–porous structures. These devices, in the future, will enable to perform repeatable, controllable, atraumatic, and repeatable injections of active factors that may affect the improvement of osteointegration and the longer survival of implants, as well as the treatment of peri-implantitis.

## 1. Introduction

**Figure 1:**
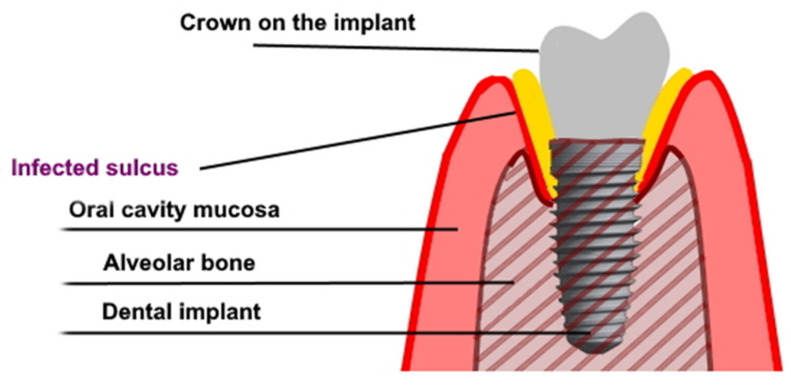
The graph shows peri-implantitis.

Dental implants, as proposed by Branemark in the 1970’s, became a recognized method of restoring missing teeth [1,2]. An implant-supported prosthesis is a restorative dental option for missing teeth. This method allows to obtain a long-lasting effect and is widely accepted by both edentulous patients and patients with partial tooth loss—consequently, improving the quality of oral health life [3,4,5]. The high acceptance of dental implants results in the constant growth of the dental implant market. Patients’ needs, as well as the sense that innovative implantological treatment may help those patients who were previously excluded from this therapy, affect the development of the market. An enormous improvement in oral health-related quality of life results from the aesthetic and functional features of dental prostheses. The dentures that are supported or retained on dental implants in many cases seem to urge doctors to use dental implants in systemically compromised patients [6]. Some publications describe the procedure for performing dental implant treatment in immunocompromised patients after solid graft transplantations, chemotherapy, or radiotherapy, as well as in patients with metabolic diseases or at an advanced age. In the cases referred to above, compromised healing and a high risk of complications (in comparison with generally healthy people) should be taken into account. These groups of patients may present a disturbed osseointegration and healing reaction as well as a different gingival microbiome, resulting in the facilitated spread of infection and development of peri-implantitis (Figure 1) [6,7,8,9]. It was found that the use of antibiotic prophylaxis is protective against early implant failures. Whenever an antibiotic prophylaxis is needed, there is still insufficient evidence to confidently recommend a specific dosage. The use of post-operative courses does not seem, however, to be justified by the available literature. In general, prophylactic antibiotics are only recommended in surgery dedicated to patients at risk of infectious endocarditis (except in non-surgical dental procedures), immuno-compromised patients, prolonged and extensive surgical interventions, surgery in infected sites, and when large foreign materials are implanted. It was found that the adjunctive use of systemic antibiotics increased mucosal recession and improved bleeding on probing in peri-implantitis [10,11,12]. Very similar mechanisms may favor osseointegration disorders and premature loss of implants in smokers and people with parafunctions. There are many reports on the implementation of implantoprosthetic treatment in patients with such conditions, and that just raises even more questions about the safety and durability of the effects of this treatment [13,14]. Dental implants are commonly placed in low-quality bone—as in patients with osteopenia or after systemic anti-resorptive treatment with bisphosphonates (BPs). Bisphosphonates inhibit osteoclast action and thereby bone resorption, and can be administered generally via oral or intravenous routes. Oral BPs are commonly used in the treatment of osteoporosis, Paget’s disease, and osteogenesis imperfecta. Intravenous BPs are used primarily for the treatment of osteolytic tumors, hypercalcemia of malignancy, multiple myeloma, bone metastases from solid tumors, and for the treatment of other tumors. Bisphosphonates used to treat those conditions might also have an effect on the clinical outcome of treatment with dental implants. Patients taking BPs may be at higher risk of implant thread exposure [15,16]. On the other hand, local bisphosphonates delivery (coating or topical) seems to enhance osseointegration in animals. From a clinical perspective, further randomized control trials with long-term follow-up are needed to confirm these results [17]. It seems to be a sensitive and insecure treatment protocol and may require some additional surgical or pharmacological interventions. The low bone density may result in an insufficient primary stabilization of the dental implant, being the main predictor for future osseointegration of the implant. On the other hand, dental implant placement in patients receiving bisphosphonates does not reduce the procedure success rate, but risk evaluation should be individualized as it may result in bisphosphonate-related osteonecrosis of the jaw (which is infrequent but may lead to very serious complications requiring a complex, multidisciplinary and unpredicted treatment) [18,19]. There is a report proving that certain medications such as selective serotonin re-uptake inhibitors and proton pump inhibitors show an association with a higher implant failure rate [20].

The general health condition is not the only factor to challenge the osseointegration of dental implants. There are many protocols concerning dental implant placement in regenerated bone or even in the bone defect site. One should mention here that vertical bone augmentation, horizontal bone augmentation, guided bone regeneration, sinus floor lift techniques, distraction osteogenesis, alveolar ridge preservation and many other techniques, which are widely used as treatments to prepare patients for implant placement or even carried out simultaneously with dental implant placement, all require more effective and quick osseointegration, which may be disturbed by many more factors than in conventional dental implant insertion protocols [21,22,23,24].

Immediate implantations do not only create the challenge for the bone volume deficiency, low primary implant stability, and the need for bone defect reconstruction, but also the increased risk of infection in the implantation site due to the socket residing bacteria. Periodontal inflammation, endodontic infections, and traumatic conditions may lead to tooth extraction as well as to the development of strains of bacteria resistant to antibiotics. Despite the above-mentioned disadvantages, new techniques and publications on immediate implantations constantly appear. The authors publish the successful cases but combine them with auxiliary procedures such as: meticulous cleaning, socket curettage/debridement, chlorhexidine or antibiotic solution irrigation, growth factors application, or the systemic administration of antibiotics and supplements such as vitamin D [25,26,27,28].

Even correctly inserted implants during the use of supported prosthetic restorations may be challenged due to peri-implant mucositis and peri-implantitis which seem to be the most frequent conditions causing dental implant loss. The weighted mean prevalence of these conditions is clinically very important and may even be as high as 47% for subject-based peri-implant mucositis [29]. The high incidence of peri-implant pathologies forces clinicians to search for novel treatments, including the site decontamination of dental implants and dental implant sites [30,31].

Systemic antibiotic shielding is effective in reducing the early loss of dental implants [32], but there are some contradictory results published by other authors who highlight significant complications of the frequent systemic use of antibiotic for dental implantology and oral surgery procedures such as allergic reactions, gastroenterological disorders and the development of microbial drug resistance. Moreover, there is a consensus about the overuse of systemic antibiotics in dental treatment [10]. Due to the shortages of systemic drug delivery, local drug administration techniques were investigated to facilitate dental implant treatment. It is suggested that the use of local chemical compound delivery systems around implants could significantly improve implant osseointegration in animal models. It is a matter of debate whether these in vivo results might have some significant effect in the human clinical setting in the longer term [33].

The aim of the study was to present the review and show the methods and devices that can be considered in the scope of the clinical practice for local drug administration to facilitate dental implant treatment. This work is a response to the ever-growing problems with osteointegration in patients with systemic diseases or infected alveolar sockets. It is associated with the demand for the use of biologically active agents to support implant treatment at its various stages (from dental implant placement, through to the long-term use of dental superstructures, to the treatment of the peri-implant conditions). In this paper, we also try to prove the effectiveness of the topical application of pharmacological biologically active agents to the implant area. To avoid some of the biases of the method that we used (narrative review), a literature search was performed in MEDLINE via the PubMed database of the United States National Library of Medicine, for articles published until 30 June 2021 using Medical Subject Heading search terms and free-text terms and in different combinations. To be included in the data screening and further analyses, studies have to: be written in the English language; be published in an international peer-reviewed journal; be human clinical trials or animal studies or in vitro studies.

## 2. Methods of Application of Bioactive Substances into Surrounding Tissues of Dental Implants

### 2.1. Drug Adhesion to the Implant Surface

According to the epidemiological data, 5 to 11% of implants fail within 15 years [34]. It is one of the reasons why implant design has been constantly modified, allowing for more predictable and stable clinical outcomes. Recently, a novel concept of using the latest achievements in nanotechnology has been introduced for dental implant treatment. The idea of modifying the titanium surface of the screw is not new. Since their introduction, constant efforts have been made to further facilitate the primary and secondary stability of the implant. Increasing hydrophilicity seems to be a logical step, since it would facilitate interaction with biological fluids at the initial stage of graft healing. The logical approach was to increase the roughness of the titanium surface, by means such as grit-blasting, acid-etching, and the anodization of spraying titanium plasma [35]. The latter brought questions of the biological effect of the increased release of titanium particles into the surrounding tissues. There were concerns about the risk of increased carcinogenesis [36]. Another approach was to modify the hydrophilic properties of the implant’s surface; however, this led to conflicting results. Buser et al. [37] observed a greater bone-implant contact percentage in individuals with modified sandblasted and acid-etched surfaces (mod-SLA) than in individuals with standard SLA surfaces (modification-regarded rinsing implant after etching in nitrous protection and storing in isotonic NaCl solution). Finally, attempts have been made to coat the implant surface with bioactive substances. Since, in ideal situations, the implant is surgically inserted into the alveolar bone, the natural choice is hydroxyapatite. There is unanimous scientific support for the superiority of such implants over traditional ones [38]. Hydroxyapatite can be used as a coating for metal implants in thicknesses from hundreds of nanometers up to hundreds of microns. As a nanomaterial, hydroxyapatite has been applied for drug delivery. The adsorption of proteins and other compounds can be adjusted by modifying their composition, electrical polarization and wettability. There are some publications on the hydroxyapatite used as the bisphosphonates carrier to treat bone loss and also a couple of other drugs to the mineral. Hydroxyapatite coating may function as a scavenger for the ions release from metal implants and thereby inhibit the adverse effects of the ion burden for the body. Hydroxyapatite is considered a safe biomaterial, unless the Hydroxyapatite coatings are very fragile and do not survive the high torque used for dental implant insertion. Nanohydroxyapatite may insidiously possess adverse effects especially when ingested by cells and may elicit excess intracellular calcium [39].

The progress achieved in the nanotechnology field allows us to utilize new concepts of implant design. There is a consensus that the nano-preparation of the implant surface improves the primary stability in the bone of the host [40]. Moreover, entering the scale of 100 nm and less brought new perspectives to the supportive treatment of individuals subjected to implantation. In 2018, the technique of nano spraying was introduced. Laboratory tests proved the effective coating of tested implants with chitosan, poly-lactic glycolic acid (PLGA) and curcumin [41]. To date, many studies have proven that PLGA shows promise as the reliable carrier for various substances, showing, on the one hand, biocompatibility and no cytotoxicity, and, on the other, the ability of a sustained release of carried agents [42]. Baghdan et al. [43] in the further study evaluated the effectiveness of implants nano-sprayed with PLGA and norfloxacin in eliminating *Escherichia coli* species in vitro. The authors observed that the antibiotic release had a two-stage dynamic. The first phase lasted 3 days and was probably associated with the detachment of the excessive amount of norfloxacin, not fully soaked into the PLGA carrier. The second phase was observed throughout the whole duration of the study (16 days), in which norfloxacin was released at a low but steady rate [43]. The spraying technique, namely, the particle fractionation effect, influenced on the content of norfloxacin in the nanoparticle coating and, therefore, also on the observed spheres of inhibition. The lower was the position of the titanium discs in the particle collector, the lower norfloxacin amount and inhibition zones [43].

Sun et al. [44] used an anodization technique to cover titanium foil with nanotubes. Then, samples were dipped in recombinant human bone morphogenetic protein (rhBMP-2) solution and coated with PLGA. Samples were placed in the wells containing sterile phosphate-buffered saline and an rh-BMP release was observed for 28 days. Observing an initial burst of the rh-BMP release lasting one week, and a much lower release for the next 3 weeks. The initial burst showed a much milder drop when a 3% concentration instead of 1% was used [40]. The achieved rh-BMP-2 secretion was high enough to promote preosteoblast adhesion and proliferation. Additionally, alkaline phosphatase activity and, therefore, osteogenic differentiation were raised significantly compared to the control [44]. Alécio et al. [45] followed a similar method. Using electrochemical treatment, they covered titanium screws with nanotubes (approximately 12 nm long and 100 nm wide in diameter), soaked them with doxycycline and coated them with PLGA. Nine implants were kept in buffer solutions and doxycycline release was observed in vitro for 30 days. Another three implants were placed in wells containing normal gingival fibroblasts, to evaluate cytotoxicity after a 3-day timespan, by the means of an MTT ((3-(4,5-dimethylthiazol-2-yl)-2,5-diphenyltetrazolium bromide) proliferation assay. The authors did not observe any cytotoxic effect against cultured cells. The drug release showed a constant 2–3-fold drop in the following periods: the first 24 h, next 2 days, next 8 days, next 17 days, and, finally, 3 days of the study. The final result was that at the end of the study, the drug release was 20–80 times lower than at the beginning of the study. This amount is sufficient for promoting osteogenic effect (neutralizing collagenase), but the question of whether that would be enough to initiate antibiotic effect, has not been answered [45]. Wang et al. [46] have studied the effectiveness of nano-zinc oxide (nZnO) in the reduction in bacterial infection in vitro and the animal model. ZnO was chosen due to its wide utilization in dentistry, and due to the approval of the substance by the FDA. Titanium and zirconium foils were covered with a complex structure of ZnO nanospheres and nanorods. Then, samples were co-cultured in phosphate-buffered saline with *Escherichia coli* and *Staphylococcus aureus* strains for 6 to 48 h. In vivo implantation was performed on male Sprague Dawley rats, where samples were implanted subcutaneously, followed by the injection of a bacterial suspension in the implantation area. Rats were sacrificed after 14 days and the sample area was examined. Results—both in vitro and in vivo—showed a bactericidal effect. However, there is a need to mention a couple of shortcomings such as the short period of observation and study design, and the lack of the incorporation of real conditions in the contest of implant insertion bacterial infection [46].

Reported implant solutions, although showing technical superiority over formerly used brands, also show common features: drug release is characterized by the hyperbolic pattern, with the peak at the first hours/days after placement, and the asymptotic decrease in the oncoming days. Same or similar observations are reported in the case of orthopedic implants [47]. While providing enough time for the healing and osteointegration process, this may not be enough for the protection against bacterial invasion. A successful short-time observation and in vitro conditions, presented clinical approaches that may encounter problems in the complex environment of the oral cavity, and even if successful, cannot bring a considerable benefit over commonly used implant designs in the longer term. Bacterial invasion into the gingival collar surrounding the implant–peri-implant mucositis is not just a matter of the healing period after the insertion or crown fixation, but is a continuous process. With the lack of a clinical attachment and reduced biological width—a natural buffer zone between the bone and gingival sulcus, in which the inflammatory process occurs—, dental implants are much more prone to develop an advanced disease—peri-implantitis. Until now, there have been no reliable, predictable and aesthetically satisfying methods of treating peri-implantitis, although such treatment in favorable conditions is possible [48]. The occurrence of peri-implant mucositis and peri-implantitis reaches 47% and 20%, respectively [29], so the problems of bacterial contamination of the implant surface, the treatment of peri-implant conditions, and management of soft- and hard-tissue deficiencies will surely become a social problem in the foreseeable future. The need for supporting home oral hygiene with additional antibacterial procedures is urgent and there is a need for an implant design that will allow drug release for several years, if not decades, after the integration with alveolar bone.

### 2.2. Application of the Drug on a Carrier to the Bone Defect during Implantation

The topical drug may be attached to its carrier by a chemical reaction or physical interaction. There are few structural forms for drug carriers used for bone regeneration in conjunction with dental implant placements: liposomes and lipid emulsions, micelles, micro/nanoparticles, films, injectable hydrogels, three-dimensional scaffolds, mesoporous materials and composites. The materials used for drug carriers should be biocompatible, non-immunogenic, and inert to the normal bone healing events. Several of natural and synthetic polymers as well as inorganic minerals have been used to deliver drugs. Natural polymers, such as gelatin, collagen, fibrin, alginate, silk, hyaluronic acid, glycosaminoglycans, and chitosan, are commonly used as carriers. Additionally, demineralized/decellularized bone matrices, being natural reservoirs of growth factors, are widely utilized [49].

Drug carriers may be the source of diverse groups of active agents that may enhance bone defect regeneration and improve dental implants osseointegration. They can be used separately or in combination with each other. We can mention bone morphogenic proteins 2 and 7 (BMP-2 and BMP-7)), the transforming growth factor β (TGF-β), vascular endothelial growth factor (VEGF), placenta growth factor (PIGF-2), platelet lysate, hormones and phytohormones, antibiotics (tetracycline, doxycycline, minocycline, clarithromycin), alendronate, simvastatin and raloxifene [50].

There are a lot of bioactive substances which have an impact on wound healing, the integration into the bone as well as on other processes that play a role in successful implantation [51]. To minimize the risk of implant failure associated, for example, with antibiotic resistance, many studies investigate different methods of therapeutic prevention. For example, one of them suggests the combination of aminolevulinic acid (ALA) with red-light photodynamic therapy against bacteria involved in infections of the oral cavity and peri-implantitis. According to this in vitro study, the effective treatment consisted of a 25 min incubation with 50% ALA followed by 5 min of a red LED. This therapy targeted both Gram-positive and Gram-negative bacteria such as *Staphylococcus aureus*, *Enterococcus faecalis, Escherichia coli, Veillonella parvula,* and *Porphyromonas gingivalis* [52]. Another in vitro study emphasizes the role of 14% doxycycline gel applied directly to the implant surface. This research showed that the adjunctive use of this antibiotic may be helpful in implant surface decontamination and the management of peri-implantitis [53]. Taking into consideration limitations of systemic therapy (for example insufficient penetration to the place of action and a low concentration of antibiotics because of it), the promising areas of research are bioactive molecules and drugs incorporated in loading systems such as thick layers of titanium oxide whose action is mainly intensified at the localization of dental implants. These substances can inhibit the formation of biofilm through blocking attachment to the surface or have direct antibacterial properties (antibiotics). Although we have a lot of groups of drugs such as antibiotics, bisphosphonates, anabolic drugs (parathyroid hormone, anti-sclerostin antibodies), estrogen, denosumab and selective estrogen receptor modulators, only a few of them have been used for implant coating. The most often applied group is bisphosphonates [54]. However, there are also new promising candidates described in the literature. Firstly, an ideal bioactive substance in implants should stimulate osseointegration and increase the bone-to-impact contact, modulating for example osteoblast function. One of them is keratin hydrogel used for dental implant coating. A pilot analysis of the sheep model indicated that keratin hydrogel promoted earlier osseointegration and increased by almost twice the percentage of the bone-to-implant contact in comparison to control [55]. Another study pointed out the role of the combination of methylenephosphonic acid surface-modified magnesium. This study proved that this combination promotes two key functions of osteoblasts: adhesion and proliferation and, in addition, an induced calcium phosphate precipitation [56]. Secondly, it is also important to provide appropriate antimicrobial properties, especially in combination with these described above. There were probes of sequential releasing of substances to combine properties of diversified agents. There were also probes of the sequential release of substances. One of the studies revealed that the early released gentamicin combined with only BMP-2, as well as with both BMP-2 and IGF-1 (insulin-like growth factor 1), showed an additive effect on metabolic activity and alkaline phosphatase activity of osteoblast-like cells in comparison to control (single coated). In this in vitro study, antibiotics prevented infections and growth factors. Subsequently, they stimulated cell proliferation and bone healing in the area of the dental implant [57]. Totarol (a natural plant-derived preservative) known for those molecules, can be also used in prophylactic. This substance extracted from *Podocarpus totara* and placed as a coating agent on a titanium dental implant surface showed significant contact killing and growth-inhibiting effects in the oral bacterial microenvironment after 4, 8, 24 and 48 h of incubation [58]. Some researchers tried to compare different coating agents amongst themselves. It is also very important to limit inflammation in the implant environment. One study indicated properties of the Ag/SiOxCy as a good antimicrobial, anti-inflammatory and stimulating osseointegration agent. However, the comparison with polysiloxane-coated implants did not show any statistically significant differences. In addition, grift-blasted and acid-etched implants had more significant osseointegration capabilities than Ag/SiOxCy [59]. Another interesting part of the research is nanoparticle studies. For example, zinc oxide nanoparticles were investigated as a coating material for dental and orthopedic implants. This study revealed that a 100% nZnO and 75% nZnO/25% nanohydroxyapatite composition had significant antimicrobial activity. Moreover, cell culture studies did not find any relevant toxicity concerning osteoblasts. Cells adhering onto the surface of the implant were also not morphologically changed [60]. The new promising material for dental implants may also be cerium oxide-incorporated calcium silicate coatings. One investigation proved that this material has relevant antimicrobial activity on *Enterococcus faecalis*, promotes osteoblastic differentiation and provides good biocompatibility [61]. Future studies concerning other pathogens and this material are needed. However, it is also essential to validate substances carefully. Many studies indicate the need for using new incorporated substances carefully with the focus on adequate doses and validated materials. It was proven on rabbits that BMP-2-coated implants had significantly lower bone-to-implant contact and the bone area surrounding the implant. This study emphasized that high doses of BMP-2 can lead to bone impairment. It points out the reason why adequate doses should be carefully validated for BMP-2 and other substances [51]. On the other hand, some studies indicate that BMP-2 increases the proliferation of cells and activity of alkaline phosphatase (which subsequently leads to more significant calcium deposition and higher expression of collagen I and osteocalcin). In vivo animal tests showed that this molecule stimulated bone formation and osseointegration in the microenvironment of the implant [62]. However, in the case of osteocalcin, another study reported that the difference of expression between the control and the BMP-2 coated implant group was not statistically significant [63]. Another controversial example is bisphosphonates (BPs). Meta-analysis based on animal studies showed that there was no significant difference in bone–implant contact in comparison between implants coated with BPs and implants without BPs [33]. Drawing conclusions from that study, the clinical use of bisphosphonates may be rather limited. However, the majority of other studies indicated that BPs have a positive impact on osseointegration [64]. In addition, a randomized trial evaluating radiographic follow-up after five years of loading showed that BP-coated implants influence on the prolonged preservation of the marginal bone. In contrast, systemic bisphosphonates therapy in people with bone metastases or osteoporosis may be associated with a higher risk of osteonecrosis [65]. Conclusions from those studies indicated that new meta-analyses in the area of bioactive substances are necessary in order to determine the new materials with the best properties for dental implants. The bioactive substances coating dental implants and their positive impact on an implant environment were described in Table 1.

### 2.3. Intra-Pocket Drug Delivery to Enhance Dental Implants

An implant in the oral cavity is challenged through the constant exposure to the bacteria that are present in saliva. In a healthy oral cavity (with proper hygiene), there are single microorganisms and their microcolonies adhering to the surfaces of hard and soft tissues. They form a so-called biofilm. It is estimated that it may contain up to 1000 different species or strains of microorganisms. Synergistic and antagonistic interactions between resident species, including potentially pathogenic species, remain in relative, dynamic equilibrium. This balance is called homeostasis. At the same time, the defense mechanisms of the human body prevent the excessive multiplication of microorganisms. The loss of homeostasis of the oral bacterial flora can lead to the development of lesions, including an excessive multiplication of some resident microorganisms of plaque and mucosa. *Staphylococcus aureus, Pseudomonas aeruginosa, Staphylococcus epidermidis,* and *Escherichia coli* have been identified as the most common organisms involved in implant-associated infections [66]. Biofilm formation on dental implants consists of four stages: (1) initially, the bacteria cells attach to the implant surface; (2) the cells aggregate and accumulate in many layers, one on top of the other; (3) the biofilm matures and (4) the cells detach from the biofilm and spread over the surface of the material to initiate a new cycle of biofilm formation [67]. Bacterial leakage takes place through the contact microfissures near the top of the implants. Manufacturers of implant systems use various methods to protect implants from bacterial marginal leakage. One of these methods is called platform switching (or platform shifting, PS). Platform switching can help to prevent crestal bone loss, which is crucial for the long-term stability and the success of implantation. PS shifts the implant–abutment junction (IAJ) inward. Signs of histological infiltration of inflammatory cells are found at a distance of 1 to 1.5 mm to the IAJ after implantation. To protect the bone from the infiltration of inflammatory cells and microbial invasion, it is necessary to determine the distance of 1 mm of healthy tissue to seal biological comparable to the surrounding natural teeth. PS at IAJ causes the inflammatory cell infiltration to shift relative to the center axis of the implant away from the adjacent alveolar bone, limiting alveolar bone resorption. The PS concept seems to limit alveolar bone reabsorption and maintain an adequate level of bone around the implant [68].

Applying drugs to the area of IAJ can additionally support this area against bacterial invasion. There are two kinds of local intra-pocket drug delivery systems: patient applicable and professional applications. Patient-applicable techniques include mouthwashes, dental pastes, chewing gums and subgingival irrigation. Professional-applicable techniques include films, fibers, gels, ointments, microspheres, nanoparticles and sol–gel transition drugs. Three different kinds of agents are applied directly to the dental implant gingival pocket: antimicrobial drugs, biofilms inhibitors, and agents modulating the patient’s immune system. Although peri-implantitis is modulated and mediated by the host, microorganisms are always responsible to initiate the inflammatory response. The pathogenic microorganism accumulation on dental implants and their components might stimulate inflammatory reactions in the peri-implant tissues and induce peri-implantitis development. The role of antimicrobial drugs is to decrease the number of microorganisms in the implant socket. The efficacy of the locally delivered antibiotic in managing peri-implantitis has shown improvements in probing depths that were significantly reduced compared with (non-treated) controls [69]. The group of antimicrobial drugs includes chlorhexidine and antibiotics.

#### 2.3.1. Antimicrobial Drugs

The simplest method is to use antiseptic preparations to eliminate the bacterial influence with agents containing chlorhexidine in a concentration of 0.1–0.2% for mouthwash used twice a day (not longer than 14 days) and gels applied to the gingival pocket around the implant. The irrigation is conducted with a syringe which is conveyed deep into the peri-implant pockets. The gel (e.g., 3% gel of chlortetracycline) can be injected around implant surfaces. The needle is positioned inside the full length of the peri-implant pocket. Slight coronal–apical–coronal movements should be performed to achieve a better distribution of the substance into the pocket. The patients cannot eat, drink, or rinse for at least 3 h because the gel has to remain in the pocket for a longer time. Chlorhexidine gel (Chlosite, GHIMAS, Casalecchio di Reno, Bologna, Italy) contains chlorhexidine in two active forms placed in xanthan (saccharide polymer), which is the carrier of 0.5% chlorhexidine digluconate and 1% chlorhexidine dihydrochloride. The xanthene carrier is responsible for the slow release of the gel components for up to two weeks, maintaining the local concentration of chlorhexidine above 100 µg/mL, which enables the elimination of pathogenic bacteria [70]. Another form of chlorhexidine administration is the PerioChip (PerioChip 2.5 mg Periodontal Insert, Dexcel Pharma Technologies Ltd., Northamptonshire, UK). It is in the form of a small 2 × 3 mm leaf and contains 2.5 mg of chlorhexidine gluconate which is cross-linked to the gelatin matrix. Gradually, the hydrolyzed matrix releases chlorhexidine until the leaf is biodegraded. The advantage of the PerioChip over other preparations containing chlorhexidine is its pharmacokinetic properties. The release of the substance from the chip takes place in a two-phase manner. During the first 24 h, 40% of the formulation is released, and then over a further 7–10 days, the chlorhexidine is gradually released. This variability is explained by the fact that at the beginning there is a so-called burst effect, which depends on the diffusion of chlorhexidine from the PerioChip. The further release is the result of enzymatic degradation. Chlorhexidine reduces the number of anaerobic bacteria (*Porphyromonas gingivalis, Bacteroides forsythus, Prevotella intermedia*) and also prevents plaque build-up [29,71,72]. The additional use of chlorhexidine rinses and gels applied to the gingival pockets around the implant enhances the therapeutic effect [73]. Chlorhexidine application methods are not precise, the concentration is diluted by saliva or the gingival pouch filtrate, the substance is unstable and should be administered frequently.

Current studies suggest more clinical benefits in the local use of antibiotics than the local use of chlorhexidine [30]. For the local application of the antibiotic, we can use tetracycline fibers, doxycycline, minocycline or metronidazole [74,75,76]. Tetracycline is a bacteriostatic antibiotic that inhibits protein synthesis. Tetracycline is effective in reducing the depth of pockets. This substance also reduces the frequency of detection of *Prevotella intermedia/nigrescens, Fusobacterium sp., Bacteroides forsythus*, and *Campylobacter rectus* [77]. The study on patients with peri-implantitis, where the 50 mg/mL dose of tetracycline was applied for 5 min after implantoplasty or air powder and followed by an autogenous bone graft or xenograft and membrane, resulted in the arrest of the disease and radiographic bone fill of the peri-implant defects [29,30]. Doxycycline is a broad-spectrum antibiotic and has antimicrobial activity against the subgingival biofilm. Doxycycline has the ability to bind to the dentin surface. This substance has a bacteriostatic effect for *Porphyromonas gingivalis, Prevotella intermedia, Campylobacter rectus* and *Fusobacterium nucleatum* at the concentration of only 6.0 μg/mL. In the case of other periodontal pathogens, the concentration ranges from 0.1 to 2.0 μg/mL [78]. The Atridox (DenMat Holdings, LLC, Lompoc, CA, USA) preparation contains doxycycline in a resorbable carrier. After insertion into the pockets, the preparation hardens, releasing active doxycycline for up to 7 days [79]. A topical application allows for a much higher concentration of doxycycline in the pouch fluid, approx. 1700 μg/mL after drug application and approx. 200 μg/mL after 7 days. In the case of oral administration of doxycycline, 200 μg/mL on the first day of taking doxycycline tablets and 100 μg/mL after 7 days. Thanks to the local application of the preparation, we can avoid the systemic influence of the antibiotic (during treatment with Atridox, the concentration of doxycycline in the serum did not exceed 0.1 µg/mL) [79]. In another study, the combination therapy of Doxycycline powder, deproteinized bovine bone mineral with 10% collagen and enamel matrix derivative in the regeneration of peri-implantitis was evaluated. The bone defects were filled with this mixture and that procedure achieved promising results [80]. The gel with metronidazole and doxycycline (20 mg/mL and 10 mg/mL, respectively) can be administered into the gingival pockets using a syringe. It is characterized by optimal final viscosity and mucoadhesive properties. Metronidazole is an antibacterial drug that is especially effective against anaerobic bacteria. Metronidazole can be effective against *Aggregatibacter actinomycetemcomitans, Prevotella intermedia, Porphyromonas gingivalis, Tannerella forsythensis, Prevotella nigrescens, Streptococcus sanguinis, Parvimonas micra,* and *Eikenella corrodens.* Metronidazole and doxycycline gel has been shown to be effective in destroying planktonic species and bacterial biofilm [78]. Minocycline is an antibiotic from a group of tetracyclines. The minocycline ointment contains 10 mg of minocycline in 0.5 g of ointment in a disposable polypropylene applicator (2% minocycline HCl). It can be used for the treatment of peri-implantitis. In the study, where minocycline was placed to peri-implant sulcus (after surgical debridement), none of the patients carried *Porphyromonas gingivalis* or *Tannerella forsythia* at 6 months. The repeated local delivery of minocycline combined with surgical treatment gives significant benefits in terms of clinical and radiographic parameters [81]. Combined intrasulcular 0.1% chlorhexidine irrigation and the local delivery of minocycline HCl gel treatment can also improve clinical and radiological indicators [82].

A huge problem is the resistance of bacteria to antibiotics. An interesting method would be the use of drugs or substances capable of sensitizing dormant persisted bacteria to existing antibiotics. Persisted bacteria are responsible for multidrug tolerance and are resistant to standard doses of antimicrobial substances. The purpose of substances against such pathogens would be to interfere with the general defense systems that protect bacteria from different antimicrobial substance. Substances would have to influence gene encoding bacterial cystathionine γ-lyase (*bCSE*). This gene is responsible for the production of H2S, which is associated with oxidative stress and protects bacteria against antibiotics. Weighing down this barrier can help in the struggle with the buildup of bacterial resistance. Thus far, three inhibitors of H_2_S production, NL1, NL2 and NL3, have been discovered belonging to the same chemotype with a central indole moiety. This requires further research [83,84].

#### 2.3.2. Biofilm Inhibitors

Biofilm inhibitors’ role is to break the biofilms or inhibit the process of biofilm formation [85]. The group of antimicrobial drugs includes chlorhexidine (described earlier), triclosan and HYBENX.

Treatment with 0.3% triclosan/2% copolymer, applied twice a day, is also useful for reducing plaque deposition. After a 6-month study, a reduction in plaque deposition and bleeding as well as over a 90% reduction in anaerobic bacteria such as *Aggregatibacter actinomycetemcomitans, Fusobacterium nucleatum, and Porphyromonas gingivalis* in the gingival pockets around the implants was demonstrated [86].

HYBENX (St. Paul, MN, USA) is another chemical desiccant substance that safely removes microbes and tissue debris from the treatment areas without impacting other areas of the human microbiome. HYBENX consists of 60% sulfonated phenolics, 28% sulfuric acid and 12% water. HYBENX’s formula of sulfonated phenols is specifically blended for the controllable and instantaneous desiccation, loosening, and coagulation of debris as it devitalizes infectious tissues. It eliminates the plaque biofilm and the matrix substructure in the oral cavity. HYBENX is based on a desiccation shock debridement technology (DSD), which consists of using a new class of a non-antibiotic cleanser to eradicate pathogens and the residual molecular matrix from infected tissue surfaces. Its selectivity allows for the reduction in bleeding in the treatment area, pain and tissue inflammation [30,87].

#### 2.3.3. Agents Modulating the Patient’s Immune System

Agents modulating a patient’s immune system may, such as doxycycline, inhibit osteoclastic activity and promote osteoblastic activity. It was found that doxycycline in sub-antimicrobial doses at 20 mg (Periostat^®^, Pharmaceutical Manufacturing Research Services, Inc. Horsham, PA, USA) is a safe and effective adjunct when taken twice daily for at least 3 months and inhibits osteoclastic activity and promotes osteoblastic activity for up to 24 months [88]. The other agents are bone anabolic drugs such as bone morphogenetic proteins, fibroblast growth factor statins, parathyroid hormone, etc., which act by targeting cell signaling pathways involved in the regulation of the osteoblastic lineage and function, enhancing alveolar bone growth at the dental implant site [89].

Bone morphogenetic proteins are a group of growth factors. BMPs have a protective effect for the early stage of bone healing, and play an important role in the regulation of osteoblast and osteointegration [36,90]. Injective gels, collagen sponge, hyaluronic acid hydrogel and microsphere hydrogel are the carrier materials for BMP [33,91]. BMP-2 is the most popular protein from the BMP family. The BMP-2 powder gel composite improves osseointegration of the dental implant. It increases the amount of new bone formation and improves bone quality and quantity. The material is placed together with implantation [90].

Some agents such as triclosan possess anti-inflammatory effects in vitro, reduce the cyclooxygenase 2 and cytokine-stimulated (interleukin 1b and tumor necrosis factor-alpha) production of prostanoids (prostaglandin E2) from monocytes in culture and inhibit bone resorption [92].

Bisphosphonates are a class of drugs that prevent the bone resorption, renewal of bone-mediated osteoclast, and increase bone density under normal conditions. Injective gels, collagen sponge or microsphere hydrogel are the carrier materials for bisphosphonates. Alendronic, zoledronic and risedronic acids are the most commonly used. Bisphosphonates have a protective effect on early formed bone and may improve the fixation of metal implants in bone [33,93]. The local delivery of zoledronic acid to gingival pockets gives significant benefits in terms of clinical and radiographic parameters [94].

### 2.4. Local Delivery Devices

Most of the available research and literature data are focused on the application of drug-release coatings to the surface of implants. However, surface engineering methods have some disadvantages for long-term treatment due to the limited time of drug release from the coating, and the inability to re-impose the coating after implantation. Additionally, these layers may be damaged during implantation, and, thus, their operation may deteriorate. Moreover, a rough surface facilitates biofilm formation which can cause an infection [95].

The easiest solution for on-demand drug release could be the use of dental implants with internal channels. Currently, there are no such solutions available on the dental market ready for patients. The only implant available on the market with internal channels is the Diva implant (Paltop Advanced Dental Solutions, Burlington, MA, USA). These channels are used to lift the sinus with saline, delivering bone substitute (Beta-TCP) to fill the sinus space, and are sealed with the inner valve screw immediately after implantation. These channels are not reused during the treatment process after closing [96]. Although there are no commercially available dental solutions, some publications show the controlled administration of drugs using different technical approaches.

For example, De Cremer et al. [97] developed an implant with an internal reservoir, which can be easily refilled. The implant is composed of a composite material that consists of porous titanium/silica (Ti/SiO_2_) obtained by powder metallurgical processing. Due to its properties, the composite material allows the diffusion of antimicrobial compounds and drug molecules from the reservoir through the porous walls of the implant into the surrounding tissues. Therefore, the formation of the microbial biofilm on the implant surface is reduced. The reservoir is closed from the top with a cover screw with the conical seal that prevents leakage of the drug solution to the oral cavity [97].

In another study, Park et al. [98] introduced a concept of an implant-mediated drug delivery system (IMDDS). The implant is hollowed and has multiple microholes that can continuously deliver therapeutic agents into the systematic body. The implant consists of a body, which is a titanium screw with multiple diffusion holes formed in the circumferential wall of the implant, the internal drug cartridge and a cover screw on top of the implant that closes the gate of the IMDDS. The specially designed nonabsorbable implant can provide sustainable drug release and eliminate the frequent use of the drug or the need for multiple painful needle injections [98].

According to recent scientific publications, the best example of a dental drug delivery device could be the open porous implant with internal channels described by Chmielewska et al. [99] in the patent application WO2021091406A1. The subject of the invention is an intraosseous dental implant used for the application of biologically active agents directly to the surrounding soft tissues and bone tissue, and their substitutes. Furthermore, the device can measure the newly formed or lost bone tissue volume immediately adjacent to the dental implant. The implant described in this patent could be created by powder in bed additive manufacturing (AM) techniques [100]. AM techniques (often called 3D printing) in medicine utilize laser or electron beams to consolidate metallic or ceramic biocompatible powders such as pure titanium, titanium alloys (Ti-6Al-4V, Ti-6Al-7Nb, etc.), ZrO_2_, or Al_2_O_3_, or other biomaterials. AM techniques can be used nowadays to fabricate dental implants with internal channels with diameters as small as around one hundred microns. The unmelted powder residuals can be removed from internal channels after fabrication by chemical and electrochemical methods. The literature shows that the chemical polishing procedure of titanium implants improves the human mesenchymal stem cell (hMSC) adhesion and proliferation [101,102]. The channels designed in the aforementioned implant could be used for the administration of the antibiotic substances to the implant surroundings at any time during patients’ treatment just after the unscrewing of the abutment and connection of the syringe feeder.

Another device that is also worth mentioning due to its local drug delivery into surrounding implant tissues is an abutment with an active substances release chamber presented by Zhang et al. [103]. Their study presents a specially designed drug-delivery abutment composed from a modified commercial piece. The device consisted of two parts: 1. a cylinder-shaped drug chamber with holes in the sidewalls and 2. the closure to seal the drug chamber. The invention allows for multiple refilling of the reservoir with antibiotics or other drugs and the controlled drug release at any stage of the patients’ treatment [103].

**Figure 2:**
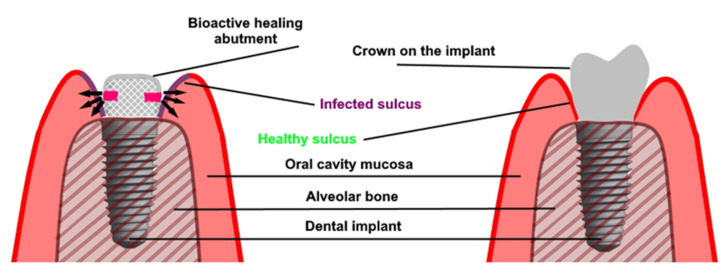
Bioactive healing abutment method of action: (**left**) treatment with bioactive healing abutment and (**right**) implant restoration after treatment.

A similar solution was presented by Iwańczyk et al. (Figure 2) [104]. The authors presented the bioactive healing abutment that was presented as a modified healing screw (patent claim p.427453). The abutment may be fixed on the implant at any time from implant placement, implant uncovering and even after a long time use of the prosthetic superstructure if peri-implantitis occur. The abutment is hollow inside and has communication holes in the lower part. These holes enable the active agents to diffuse directly to the gingival sulcus of the dental implant avoiding the drug dissemination into the whole oral cavity. The bioactive healing abutment may be atraumatically reloaded with any content such as antimicrobial agents, anti-inflammatory or anesthetic drugs, or even growth factors [54].

## 3. Discussion

The local application of drugs in the area of a dental implant is most often aimed at the soft tissue or alveolar bone regeneration or combating inflammation, often of bacterial etiology, in particular peri-implantitis. To achieve efficacy and a lasting therapeutic effect in the treatment of these pathological conditions, it is necessary to achieve a strictly defined therapeutic pharmacological agent concentration [33,54].

Alveolar bone regeneration is dependent on both endosteal and periosteal bone formation and, as found by Colnot [105], the periosteum, endosteum and bone marrow are major sources of skeletal stem cells/progenitors and contribute differently to osteogenesis and chondrogenesis during bone repair. The periosteal injuries are healed by endochondral ossification, whereas endosteal/bone marrow injuries are healed by intramembranous ossification without chondrogenesis. The regeneration process can be summarized into four critical stages: the inflammation phase (hematoma formation and skeletal stem cell recruitment), the soft callus phase (neo-angiogenesis and cartilage/bone formation), the hard callus phase (cartilage resorption and/or active bone deposition) and the remodeling phase. All these phases are very specific and sensitive to different molecular actions and bioactive substances [89]. Therefore, the use of only one biological or pharmacological agent bound, e.g., to the surface of a dental implant, may not be sufficient to facilitate and enhance impaired osteogenesis and osteointegration. What is more, the process of bone healing from injury to the first radiological symptoms of the formation of the compact bone bridge in humans is no less than 10–12 weeks, not including the bone remodeling phase. The systematic enhancement with a parathyroid hormone or anti-sclerostin antibody (romosozumab) may shorten the time needed for fractured bone healing but not less than 8–9 weeks and may require multiple applications of the active agent which is not possible for contemporary dental implants [54,106]. All these clinical observations are coherent with the physiology of bone defect and fracture repair. Signals that are not yet completely understood attract osteoclasts, multinucleated bone-resorbing cells, to sites that become a bone-remodeling unit. When the resorption of bone by osteoclasts in that remodeling unit is completed, a process that takes 3 to 5 weeks, the resorbed surface attracts osteoblasts, mononuclear bone-forming cells that fill the basic multicellular unit with a new matrix. The actions of the osteoblasts and the subsequent completion of the remodeling sequence by mineralization of the matrix takes 3 to 5 months [95]. These clinical and laboratory observations imply the long-term (even a few months) need for action of all active agents to enhance the bone healing process and dental implant osseointegration. Unfortunately, the carriers for active factors used for bone healing enhancement known to this day are characterized by a rapid release of them immediately after implantation and a steep pharmacokinetic curve. We observed a rapid burst release such that only 5% of the loaded BMP-2 remains after 14 days after application. The initial high burst of BMP is in contrast to the gradual increase in levels of endogenous BMPs (BMP-2, BMP-4, BMP-6 and BMP-7) that are produced during normal bone healing that peak at day 21. The same release profiles, unfavorable from the pharmacokinetic point of view, from their carriers have other biological agents used to improve the healing and regeneration of bone tissue by acting on BMPs, fibroblast growth factor 2 (FGF-2), hedgehog proteins, the parathyroid hormone (PTH), PTH-related protein, transforming growth factor β (TGF-β), protein wingless morphogenetic factors, Wnt proteins and Wnt signaling antagonists [33,89,107,108].

The peri-implantitis is a chronic condition affecting the gingiva and the bone commonly from the coronal part of the dental implant. The management of peri-implantitis comprises conservative (non-surgical) and surgical techniques. There are lots of strategies for peri-implantitis treatment, including detoxifying the implant surfaces, including mechanical methods, chemicals, laser and photodynamic therapies, but the results of some of them are contradictory and uncertain over the long term [29,30,109]. Some studies in the literature reveal a better osseointegration process in implants when treated with photobiomodulation and ozone therapy compared with control groups [110]. Ozone showed great potential for the management of peri-implant mucositis [111]. Gaseous ozone showed selective efficacy to reduce adherent bacteria on titanium and zirconia without affecting the adhesion and proliferation of osteoblastic cells [112]. The most modern directions of development of surface engineering include additive methods. Direct metal laser sintering (DMLS) has been appreciated in the field of oral implantology due to the possibility of building three-dimensional (3D) components from titanium powders with minimal or even no post-processing requirements. It was proved, that the DLMS process associated with chemical treatment modifies the in vitro biofilm profile. What is more, this process reduced the proportion of red complex and decreased the total counts of *Porphyromonas gingivalis* and it also favored (to a lesser extent) *Parvimonas micra*, which might be related to peri-implant disease [113]. The point is that peri-implantitis is a ubiquitous disease and is directly influenced by the passage of time and surely, the more time passes, the more implants will be affected by peri-implantitis. On the other hand, neither the treatment strategy of peri-implantitis treatment seems to be effective when used alone and requires the use of support techniques that are often stretched over time. It is due to the specific etiology factors causing this condition. These factors are sometimes non-changeable such as the implant insertion technique and position, implant surface, primitive implant–abutment connection, soft tissue conditions, prosthetic superstructure design or the disturbing foreign body equilibrium between the host immune system and the implant device [114,115].

All of these results indicate the need for recurrent local antimicrobial and anti-inflammatory drug administration in the strict implant area that should be very precise in the dose and be characterized by a high bioavailability and the long, controllable time of action. The point is that almost all local drug delivery for dental implant systems do not provide these features or do not pass clinical studies. The negative aspect of these anti-peri-implantitis pharmacological strategies based on the local peri-implant drug delivery is the lower implanted material regeneration potential and the need for a surgical procedure to apply an antibiotic. Additionally, an important downside to the use of drug-exuding scaffolds is their price and high specificity, expressed by the lack of modification possibilities of drug type and dose. Furthermore, they are restricted to one-time use [33,54,104,116].

The available literature lacks a literature review on the topical administration of biologically active agents to enhance dental implants. The proposed literature review shows new directions of research, their degree of advancement on the way to implementation to the human clinic. The weaknesses of this work are the scarcity of literature reports on this topic. Most of the available publications present pre-clinical studies, some of them difficult to adapt directly to the clinic. The next step, that can organize the knowledge in this field, is to conduct a systematic review in several thematic areas described in this paper. This review should be treated as an inspiration for the development of clinically effective methods and appliances for the topical application of drugs to enhance dental implants. Reporting bias is a widespread phenomenon in the medical literature. The narrative review is burdened with errors related to the nature of the publication type. It is necessary to take into account the following problems: publication bias, time lag bias, multiple (duplicate) publication bias, location bias, citation bias, language bias and outcome reporting bias [117].

## 4. Conclusions and Future Research Outlook

Local drug delivery near the implant is necessary especially in the case of using this method in patients with systemic diseases and in cases of infected alveolar sockets as well as bone deficiencies and the expectation of the longevity of the result. The currently used methods usually allow for the administration of only a single treatment agent (adhesion to the surface of the implant or surgical administration to the vicinity of the implant) or there is no possibility of their precise dosing and secretion (gingival pocket). A few reports on repeatedly used drug applications are incomplete (lack of precise clinical studies) and insufficient (too few methods). Therefore, there is a need for research in the field of inventions (medical device, or changing the structure of the implant from solid to porous) enabling in the future repeatable, controllable, atraumatic and repeatable injections of active factors that may affect the improvement of osteointegration and longer survival of implants as well as the treatment of peri-implantitis.

## Figures and Tables

**Table 1:** New and old bioactive substances coating dental implants and their positive impact on an implant environment.

Substance	Study	Results
Ag/SiOxCy	Smeets et al. (2017)	↓ growth of different bacteria ↓ inflammation ↑ osseointegration
Bisphosphonates	Najeeb et al. (2017), Abtahi et al. (2016)	↑ osseointegration ↑ preservation of marginal bone
BMP-2	Ramazanoglu et al. (2013) Yang et al. (2017)	↑ bone formation ↑ osseointegration ↑calcium deposition and bone density
Cerium oxide-incorporated calcium silicate coating	Qi et al. (2015)	↓ growth of *Enterococcus faecalis* ↑ osteoblast differentiation ↑ biocompatibility
Gentamicin + BMP-2 + IGF-1	Strobel et al. (2011)	↑ killing of bacteria ↑ metabolic activity and alkaline phosphatase activity (calcium deposition) ↑ osteoblast differentiation and cell proliferation ↑ chondrogenesis and synthesis of collagen
Keratin hydrogel	Campbell et al. (2014)	↑ osseointegration ↑ bone to implant contact
Methylenephosphonic acid surface-modified magnesium	Zhao et al. (2015)	↑ adhesion and proliferation of osteoblasts ↑ calcium phosphate precipitation
Totarol	Xu et al. (2020)	↑ contact killing of bacteria ↓ growth of bacteria
100% nZnO or 75% nZnO/25% nanohydroxyapatite	Memarzadeh et al. (2014)	↓ growth of different bacteria

## Data Availability

The data presented in this study are available on request from the corresponding author. The data are not publicly available due to privacy restrictions.

## Abbreviations

ALA	aminolevulinic acid
BMP-2	bone morphogenetic proteins 2
BMP-7	bone morphogenetic proteins 7
BPs	bisphosphonates
DMLS	direct metal laser sintering
DSD	shock debridement technology
FGF-2	fibroblast growth factor 2
hMSC	human mesenchymal stem cells
IAJ	implant–abutment junction
IGF-1	insulin-like growth factor 1
IMDDS	implant-mediated drug delivery system
nZnO	nano-zinc oxide
mod-SLA	modified sandblasted acid-etched surface
MTT	(3-(4,5-dimethylthiazol-2-yl)-2,5-diphenyltetrazolium bromide
PIGF-2	placenta growth factor
PLGA	poly-lactic glycolic acid
PS	platform switching or platform shifting
PTH	parathyroid hormone
rhBMP-2	recombinant human bone morphogenetic protein
standard SLA surface	modification-regarded rinsing implant after etching in nitrous protection and storing in isotonic NaCl solution
TGF-β	transforming growth factor β
VEGF	vascular endothelial growth factor
